# The Common Concept of Anticooperativity Among Molecules Is Fundamentally Flawed, Based on Novel and Unified Molecular-Wide and Electron Density (MOWeD) Concept of Chemical Bonding

**DOI:** 10.3390/molecules30091944

**Published:** 2025-04-27

**Authors:** Ignacy Cukrowski, Stéfan Zaaiman, Shahnawaz Hussain

**Affiliations:** 1 Department of Chemistry, Faculty of Natural and Agricultural Sciences, University of Pretoria, Lynnwood Road, Hatfield, Pretoria 0002, South Africa; u17047693@tuks.co.za (S.Z.); shahnawazh7084@gmail.com (S.H.); 2 Department of Computer Science and Engineering, Indian Institute of Technology, Kharagpur 721302, India

**Keywords:** cooperativity, anticooperativity, water clusters, molecular-wide and electron density (MOWeD)-based approach, FALDI electron density decomposition, FAMSEC

## Abstract

A non-linear (non-additive) increase in stability of hexamers follows an increase in the total number of (i) **aad** (a double proton acceptor) plus **add** (a double proton donor) waters commonly linked with anticooperativity and (ii) the total number of intermolecularly delocalized electrons (^intermol^*N*^deloc^) in the 3D space occupied by a hexamer. Subsequently, we obtained nearly a perfect linear correlation between increase in the cluster stability and ^intermol^*N*^deloc^. Individual water molecules that act as either **aad** or **add** (i) delocalize the largest number of electrons throughout a cluster; (ii) are involved in the strongest attractive, hence energy-stabilizing intermolecular interaction with the remaining five waters; (iii) have the most significant quantum component of the intermolecular interaction energy and (iv) relative to six non-interacting water molecules, stabilize a hexamer the most, as quantified by a purposely derived *mol*-FAMSEC energy term. Clearly, the **all-body** approach used in the unified, molecular-wide and electron density (MOWeD)-based concept of chemical bonding contradicts the commonly accepted view that **aad** and **add** water molecules are involved in anticooperativity in 3D water hexamers. Consequently, we propose here a general definition of cooperativity that should be applicable to any *n*-membered molecular cluster, namely *the quantifiable, classical physics- and quantum-based cooperativity phenomenon is synonymous with the intermolecular **all-body** delocalization of electrons, leading to the increase in stability of individual molecules on an n-membered cluster formation.*

## 1. Introduction

Water clusters and, in particular, non-additivity in strength and other properties of intermolecular H-bonds have been studied for many decades by exploring underlying processes coined as cooperativity [1,2,3,4,5,6,7,8,9,10,11,12,13,14,15,16,17,18,19,20,21,22,23,24,25,26] and anticooperativity [26,27,28,29,30,31,32,33,34,35] between water molecules. Actually, interest in properties of water clusters continues to date due to importance of water in, e.g., supporting life, and a few very informative reviews have been published on cooperativity in water clusters [9,10,17,20,36,37,38]. Considering anticooperativity, this concept was introduced well after cooperativity, and it is always investigated and discussed in conjunction with cooperativity. Although it is not a subject of this contribution, it is important to stress that the concepts of cooperativity/anticooperativity are also explored in many different kinds of molecular clusters where individual molecules are involved in intermolecular H-bonding [29,39,40,41,42,43,44,45,46].

When water chains or cyclic homodromic structures are formed, then all water molecules are of **ad** character, i.e., being arranged in the (proton acceptor, **a**)–(proton donor, **d**) configuration···O–H···O–H···O–H···. For the **ad** configurations of water molecules in clusters, non-additivity presents itself as mutual enhancement of H-bonds, leading to the binding energy being greater than that computed for a dimer. The process of the enhancement of bonding (binding energy) was named ‘cooperativity’ between water molecules [1].

Ojamäe and Hermansson [26] arranged five water molecules in the form of tetrahedron using dimers as building blocks. This resulted in the central water molecule acting as a double proton acceptor (**aa**) and a double proton donor (**dd**), meaning that the central water molecule in the tetrahedron had **aadd** functionality. Using OH frequencies computed for tetrahedron, they concluded that, for the **aadd** configuration, ‘*the nonadditivity works in the opposite direction (“anticooperativity”)*’ [26] when compared with single acceptor–single donor **ad** configurations in chains or homodromic cyclic pentamer. Such non-additivity that diminishes bonding typically is described as ‘anticooperativity’, but it is clear that they were not in favor of this nomenclature. Instead, they proposed a concept of ‘*strict cooperativity*’ that, according to them, is indisputably observed for the energies and the frequencies in chains and homodromic cyclic water pentamer (in general, in cyclic homodromic water clusters). Finally, they concluded that ‘*Water molecules in a tetrahedral coordination display cooperativity, but not strict cooperativity*’ [26]. Steiner stressed that, because hydrogen bonds may not only enhance but also reduce the strengths of each other, a change of orientation of H-bonds from homodromic to antidromic arrangement leads to *local anticooperativity* [36].

Intuitively, the concept of cooperativity among water molecules can be understood as contributions made by 2-, 3-, and (many-) body energy terms that enhance the strength of H-bonds in water clusters. Realizing that the same physics and quantum chemical rules apply to all possible sizes and shapes of water clusters, it is difficult to explain the concept of anticooperativity. To this effect, how do water molecules ‘decide’ on the mode of action, by either enhancing or diminishing bonding using the same 2-, 3-, and *n*-body energy terms? One must realize that neither cooperativity nor anticooperativity are well-defined quantum properties but rather are used to loosely describe ‘positive’ or ‘negative’ additivity of energy terms on a cluster formation. For instance, anticooperativity is descriptively defined as ‘*Negative cooperativity between binding sites on the same (macro)molecule by which binding of a ligand to one site makes the binding of a second ligand more difficult*’ in the Oxford Dictionary of Biochemistry and Molecular Biology [47].

Recently [48], using the **all-body** and molecular-wide and electron density (MOWeD)-based approach [49], we proposed a new and quantifiable concept of cooperativity. According to our recent interpretation, the origin of **all-body** cooperativity is rooted in physics- and quantum-based processes of electron (*e*) delocalization between water molecules. In other words, cooperativity is the intermolecular **all-body** *e*-delocalization leading to the non-additive increase in stability of a water molecule in clusters with an increase in their size.

Taking into the account the above and realizing that (i) delocalization of electrons is a measurable quantum process that always leads to a decrease in the energy of a system and (ii) the formation of water clusters from, e.g., water monomers, occurs spontaneously, it would follow that the process of anticooperativity would require a physical process reversing or obstructing sharing of electrons. This supposition leads us to the following hypothesis that constitutes the focus of the present contribution.

**Hypothesis 1.**

*From the fact that:*

1. * Three-dimensional six-water clusters discussed in this work are more stable than two-dimensional cyclic homodromic water hexamer, and the latter is restricted to **ad** configurations showing only positive, non-additivity in the strength of H-bonds, i.e., classical cooperativity;*
2. *Prism, being the most stable 3D six-water hexamer, does not have water molecules of **ad** configurations (there are three **aad** and three **add** waters),*

*it would follow that the double-acceptor (**aad**) and double-donor (**add**) water molecules must contribute to cluster stability more than **ad** configuration*.

If the above hypothesis holds, then the anticooperativity cannot be supported and hence must be rejected. Three-dimensional six-water clusters (bag, book, cage, and prism) are used here as model systems as they all:

i. are more stable than the six-water cyclic hexamer; notably, they are the smallest 3D clusters that are more stable than their cyclic counterparts;
ii. have water molecules with **aad** and **add** configurations, and these motifs are observed in each of the ice polymorphs [2].

One must stress that the ***all-body*** MOW*e*D-based protocol treats all atoms as a constellation of nuclei that spontaneously drive electron density (ED) to the global minimum of electronic energy of a molecular system. This approach is in accord with Bader’s view that there are ‘*only two forces operative in chemistry, the Feynman force exerted on the nuclei and the Ehrenfest force exerted on the electrons*’ [50].

To probe the above hypothesis, we will make use of recently developed tools incorporated in the all-body MOW*e*D-based approach that will provide qualitative and quantitative interpretation of cooperativity/anticooperativity, namely:

i. The Fragment, Atomic, Localized, Delocalized and Interatomic (FALDI)-based electron density decomposition scheme [51,52,53]. FALDI meets the all-body requirement perfectly, as it treats atoms’ and atom pairs’ *e*-distributions holistically over the entire 3D space occupied by a molecular system.
ii. The Fragment Attributed Molecular System Energy Change (FAMSEC) family of methods [54,55] is used to identify molecular fragments that either drive or obstruct a chemical change the most. FAMSEC also meets the all-body requirements, as the quantified energy contributions made by fragments are harvested from the entire space occupied by a system.

## 2. Theoretical Background

### 2.1. The FALDI Density Decomposition Scheme

FALDI is a unique electron density decomposition scheme developed recently [51,52,53]. The usefulness and power of FALDI were already demonstrated in the study of numerous bonding and non-bonding interactions in a number of systems [49,51,52,53,56,57]. It is unique, because one can compute, besides classical electron populations, the **exact** localization and **exact** delocalization electron counts that are quite different from localization and delocalization indices generated within the QTAIM formalism [58]. Another unique and very instructive feature of FALDI is its ability to generate 3D distributions of localization and delocalization patterns that can be visualized in real space.

In this contribution, we will focus on useful aspects of FALDI that are directly applicable in the study of cooperativity and anticooperativity in 3D water clusters. Readers interested in the full theoretical description of FALDI are referred to our recently published work [48] and references therein.

The FALDI-based electron density (ED) decomposition scheme provides (i) *atom*–ED distribution, (ii) the contribution made by electrons localized to a selected atomic basin Ω_A_, i.e., *loc*–ED distribution, and (iii) the contribution made by electrons delocalized between all unique basin-pairs (Ω_A,_ Ω_B_), i.e., *deloc*–ED distribution. All these distributions are additive and can be conveniently combined to form fragment F distributions by accounting for selected atomic basins’ contributions. From this follows that the total electron population of a *k*-atom fragment, e.g., *3*-atom fragment of a molecular system, such as a water molecule W in a cluster, *N*^total^W, can be decomposed as

(1) $$N^{\mathrm{total}}W=N^{\mathrm{self}}W+N^{\mathrm{deloc}}(W,R)$$

where the fragment’s electron population is made of two components, namely electrons that can be found only in the space occupied by a water molecule W; this count is called a ‘self’-fragment electron population, *N*^self^W, and electrons delocalized with the remaining five water molecules in a cluster treated as a fragment R, *N*^deloc^(W,R). It is important to realize that, in the case of multi-atom fragments, such as water molecules, the ‘self’ fragment electron population *N*^self^W is made of two kinds of electrons: (a) electrons localized to each atom A, *N*^loc^(A) of a water molecule treated as a fragment W. Hence, for all atom-localized electrons in a water molecule, we can write *N*^loc^W = ∑ *N*^loc^(A), where A ∈ W; and (b) electrons delocalized amongst atoms of the fragment W due to intra-fragment delocalization processes taking place, *N*^deloc^(W,R). Notably, the two delocalization terms, *N*^deloc^(W,R) and *N*^deloc^(W,R), are fundamentally different, as the latter represents inter-fragment electron delocalization, and it counts electrons that can be found in both fragments, W and R. The term *N*^deloc^(W,R), is calculated by summing up only the contributions made by atoms within the *k-*atom (here 3-atom) fragment W to diatomic DIs involving all other atoms in the molecular system (here water cluster):

(2) NdelocW,R=∑A∈Wk∑B∈Rn−kDIA,BA

Three-dimensional placement of water molecules in each cluster is unique, meaning that each water moleculeW finds itself in a different molecular environment. This, in turn, must have an impact on the number of electrons each W is delocalizing to the remaining waters in a cluster. Due to the additivity of FALDI-computed terms, one can compute the total number of delocalized electrons in each cluster by summing up the *N*^deloc^(W,R) terms obtained for individual water molecules.

It might be very useful and informative to investigate electron delocalization patterns throughout a molecular system by computing delocalized electrons counts between molecular fragments. As an example, for the *k*-atom fragment F and *l*-atom fragment H, we can write

(3) $$DI\left(F,H\right)=\sum_{A\in F}^{k}\sum_{B\in H}^{l}DI\left(A,B\right)$$

where contributions made by individual atoms are accounted for, as DI(A,B) = ^A^DI(A,B) + ^B^DI(A,B), notably DI(F,H) = *N*^deloc^(F,H) + *N*^deloc^(H,F) and, in most cases, $N^{deloc}\left(F,H\right)\;\neq$ $N^{deloc}\left(H,F\right)$. This means that $N^{deloc}\left(F,H\right)$ measures the degree to which electrons from the *k*-atom fragment F are delocalized within the *l*-atom fragment H. By analogy, a similar process applies to the *N*^deloc^(H,F) term. On the other hand, DI(F,H) measures the total electron count due to the delocalization of electrons between atoms of both fragments, i.e., the count of electrons **shared** by the two fragments. Naturally, Equation (3) is useful to compute the total number of electrons shared by any water pair in a cluster.

### 2.2. A Fragment Attributed Molecular System Energy Change (FAMSEC) Protocol

Many molecules, e.g., polyamines, show an incredible flexibility and are able to re-arrange their constellations of nuclei and change the associated electron density distribution in 3D space spontaneously leading to a large number of structural conformers of the same molecule. This can be seen as an intramolecular event. Moreover, many molecules can rearrange their relative placement in 3D space, leading to a countless number of molecular clusters, such as water clusters. This can be seen as an intermolecular event. However, what drives a change and what is the energy contribution made by specific atoms, molecular fragments, etc. when a molecular system undergoes a change from its initial (*init*) to a final (*fin*) state? From a need of understanding and quantifying changes taking place throughout a molecular system on a chemical event, the concept of FAMSEC [54] was born.

Complex chemical events take place along the reaction energy profile (REP). A dedicated REP-FAMSEC protocol was designed [55] to explore a reaction mechanism leading to new chemical products. The FAMSEC method [54] and its ‘derivatives’, namely REP-FAMSEC [55,59] and π-FARMS (Preorganized-Interacting (π) Fragments Attributed to Relative Molecular Stability [60]), pinpoint *n*-atom molecular fragments G (a molecule might also be treated as G) that drive/obstruct a chemical change the most and have also been employed to investigate non-covalent interactions in a number of systems [61].

Many energy terms can be defined within the FAMSEC method and its derivatives that make use of the exhaustive energy partitioning schemes implemented in QTAIM [58] and IQA [62,63], where entire molecular space is occupied by atoms without voids between them. This assures that harvesting of data from all corners of a molecular system, as required by the MOW*e*D-based approach, can be met. Making use of the IQA-defined principle energy components of a molecular system, the *mol*-FAMSEC term was designed to quantify energy contribution made by any *n*-atom fragment G on a chemical event. To account for molecular-wide contributions made by all atoms of a molecular system, the *mol*-FAMSEC term is defined as

(4) $$\mathrm{mol}\text{-}\mathrm{FAMSEC}=E_{\mathrm{self}}^{G}+E_{\mathrm{int}}^{G}+E_{\mathrm{int}}^{G,R}$$

The first two terms in Equation (4) constitute the *loc*-FAMSEC = $E_{\mathrm{self}}^{G}$ + $E_{\mathrm{int}}^{G}$ term that quantifies energy change that is localized (*loc*) to the 3D space occupied by *n*-atoms of a selected molecular fragment G. This involves (a) a change in self-atomic energies of atoms constituting G ($E_{\mathrm{self}}^{G}$) and (b) the change in all unique intra-fragment diatomic interaction energies, i.e., the $E_{\mathrm{int}}^{G}$ term, on the transformation of a molecular system from the initial (*init*) to final (*fin*) state. The last term in Equation (4), $E_{\mathrm{int}}^{G,R}$, accounts for changes in the strength of all diatomic interactions between atoms of G and all remaining atoms treated as a molecular fragment R. Finally, it is important to stress that all terms in Equation (4) quantify a specific energy contribution to the electronic energy of a molecular system on the *init* →*fin* chemical event.

## 3. Results and Discussion

**Figure 1:**
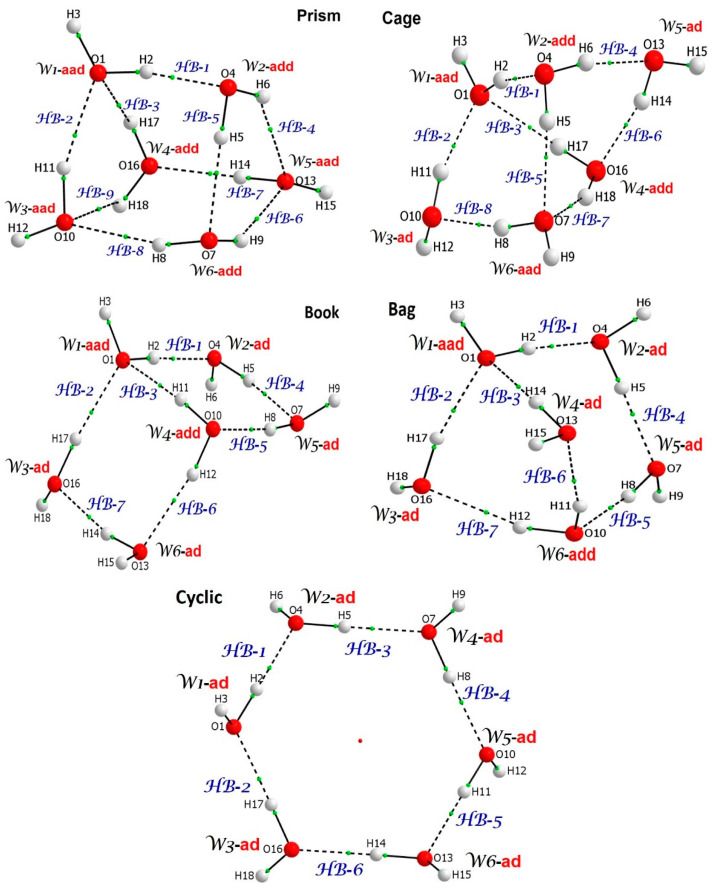
Molecular graphs of water hexamers investigated in this work showing atoms’ numbering, numbering of water molecules (Wn) and numbering of hydrogen bonds (HB-n). Functionality of water molecules, in terms of accepting (**a**) or donating (**d**) a proton, is also indicated.

Water clusters used to study cooperativity/anticooperativity are shown in Figure 1. Each water molecule is considered as a three-atom molecular fragment of a six-membered water cluster named the W*n*-**mode,** where ‘**mode**’ stands for the **ad**, **aad** or **add** mode of action (functionality) a water molecule is involved in. Moreover, each three-atom fragment representing a classical intermolecular H-bond is denoted as HB-n. All energy terms were computed using non-interacting separate water molecules that served as a reference state throughout the entire investigation.

### 3.1. Validation of B3LYP-Computed Relative Stabilities of Water Hexamers

Three-dimensional density distribution varies with the level of theory to some degree; hence, somewhat different electronic energies (*e*-energies) are expected for each cluster. Our focus, however, is on trends in values of relative electronic energies of hexamers under investigation. As exemplified by the data in Table 1, the relative energies (Δ*E* = *E*(hexamer) – *E*(prism)) computed at a very high level of theory, namely CCSD(T)/CBS [64], compare very well with data obtained at somewhat lower levels, e.g., MP2(full)/aug-cc-pVTZ [65] and CCSD(T)/aug-cc-pVTZ [66].

**Figure 2:**
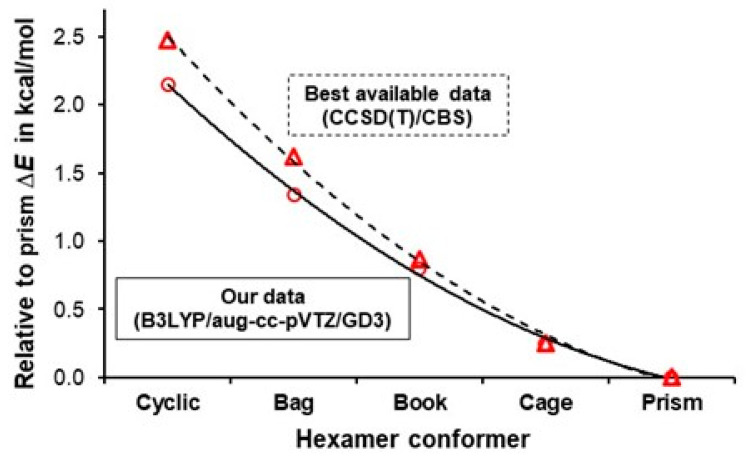
Trends in relative stability of hexamers obtained at the B3LYP/aug-cc-pVTZ/GD3 level of theory employed in this work (circles with the solid trend line), and best available data (triangles with the dashed trend line) obtained at the CCSD(T)/CBS level. Δ*E* = *E*(hexamer) − *E*(prism).

Most importantly, however, Δ*E* values computed at the significantly lower level used in our studies, i.e., at the B3LYP/aug-cc-pVTZ level with the empirical GD3 Grimme’s dispersion correction, follow exactly the same trend as obtained at the higher levels (Figure 2).

One can also see that our Δ*E* values differ from those obtained at the higher levels by a small fraction of kcal/mol (see Table 1 and Figure 2). From that, we conclude that the FALDI- and FAMSEC-based analyses performed on optimized, in this work, hexamers should provide scientifically sound and highly reliable pictures and conclusions.

### 3.2. A Cooperativity-Driven Decrease in E(hexamer) Relative to E(6H_2_O)

Regardless of the chemical system considered, classical interactions and quantum effects involving all atoms govern a relative 3D placement of atoms. This means that the universal laws of classical and quantum physics/chemistry will spontaneously drive the placement of nuclei and associated distribution of ED that is characteristic for local/global electronic energy minima. Clearly, these are **all-body** processes leading to unique constellations of atoms constituting a molecular system at each energy minimum on the potential energy hypersurface. Each constellation of nuclei generates a specific, like a fingerprint, 3D ED distribution from which all the properties of a molecular system can be derived, as stressed already over a half of century ago by Hohenberg and Kohn [67], more recently by Bader [58] and in a very recent review by Koch et al. [68].

From the above, it follows that the electronic energy *E* of a molecular system and its fragments, like water molecules constituting a water cluster, as well as some components of *E,* can be used to explore physical/quantum properties leading to or linked with cooperativity or anticooperativity using the MOWeD-based approach. Recently [48], we postulated that a non-linear increase in the stability of a water molecule, i.e., a non-linear decrease in averaged *E* of a water molecule with an increase in the number of water molecules in homodromic rings, can be used to quantify *cooperativity phenomenon* taking place in cyclic water clusters.

It is evident that our definition of cooperativity phenomenon [48], i.e., ‘the intermolecular **all-body** *e*-delocalization leading to the non-additive increase in stability of a water molecule in clusters with an increase in their size’, cannot be applied directly to clusters studied here, because all of them are six-membered 3D structures. However, it can be re-written to a more general form, namely the cooperativity phenomenon is synonymous with the intermolecular **all-body** *e*-delocalization, leading to the increase in stability of water molecules on a cluster formation.

**Figure 3:**
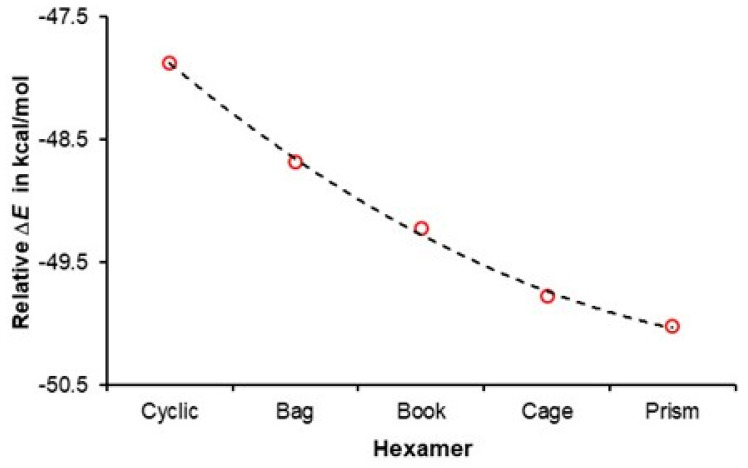
Relative to six free, non-interacting water molecules, an increase in the stability of indicated water hexamers. Δ*E* = *E*(hexamer) − *E*(6H_2_O).

From the above, it follows that an *n*-membered water cluster should have lower electronic energy when compared with separate (non-interacting) *n* water molecules. A trend in Figure 3 shows that (i) all hexamers have much lower energy than six non-interacting water molecules, and (ii) all 3D hexamers are more stable than the cyclic homodromic ring. Moreover, a non-linear decrease in electronic energy reaches a minimum value for the prism, and this strongly suggests that the cooperativity phenomenon is strongest in this hexamer.

**Figure 4:**
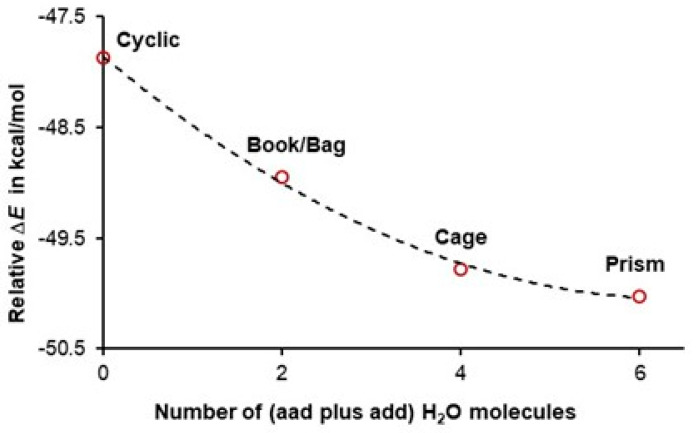
Relative to six non-interacting water molecules, an increase in the stability of indicated hexamers with an increase in the number of double-acceptor and double-donor water molecules in a cluster. Δ*E* = *E*(hexamer) − *E*(6H_2_O).

It has been accepted for decades that the double functionality of water molecules (**aad** and **add**) implies that they are involved in anticooperativity. A change in the electronic energy of a six-membered water cluster with the total number of double acceptors and donors is shown in Figure 4. Remarkably, the trend seen in Figure 4 is a ‘copy’ of that seen in Figure 3, and it strongly suggests that, the larger the number of water molecules acting as double donor/double acceptor, the more significant the cooperativity among water molecules. The combined trends in Figure 3 and Figure 4 provide the first (i) support for our hypothesis 1 stated in the Introduction and (ii) evidence against the concept of anticooperativity, as all water molecules in prism act as either a double acceptor or double donor.

### 3.3. Quantifying Cooperativity

Considering our definition of cooperativity and looking at the data presented in Figure 3 and Figure 4 leads us to the second hypothesis that, if confirmed, would not only support our initial hypothesis but also would provide quantified explanation on how double-acceptor/-donor molecules stabilize clusters more than water molecules with **ad** functionality.

**Hypothesis 2.**

*Water molecules acting as a double donor (**add**) and double acceptor (**aad**) must delocalize a larger number of electrons throughout a cluster than **ad** water molecules, and this makes the six-membered 3D clusters more stable than the homodromic cyclic hexamer.*

To test our **hypothesis 2**, we make use of the FALDI-defined electron delocalization indices. To gain a full description without any presumptions made, all-body contributions made to the total final property of a system by all water molecules and each atom are accounted for. Recall that FALDI expands the concept of ED sharing to the entire 3D space occupied by a molecular system, such as a water cluster, and delocalization indices can be computed for all atom pairs and atoms, as well as any molecular fragment.

It is important to realize that, due to very different distribution of water molecules in 3D hexamers, each water molecule finds itself in a unique environment that must have an impact on the number of delocalized electrons. To this effect, the bag and book clusters have one **aad,** one **add** and four **ad** water molecules. Even though the number of water molecules with a specific functionality is the same, we noted that the number of intermolecularly delocalized electrons ^intermol^*N*^deloc^ computed for **aad** (2.188*e*) and **add** (2.075*e*) waters in the bag is slightly larger than in the book hexamer (2.100 and 2.048*e*, respectively). Also, the number of delocalized electrons by **aad** configuration appeared to be slightly larger than that by the **add** configuration in the cage hexamer.

To account for the impact of the molecular environment, and realizing that we are dealing with rather a small sample of hexamers (a total number of 80 conformers was reported by Xantheas [19]), we decided to compute the average number of ^intermol^*N*^deloc^ for each **ad**, **aad** and **add** functionality observed in the 3D hexamers studied here. We found that, on average, **ad**, **aad** and **add** water molecules in the 3D hexamers delocalize 1.54 ± 0.12, 2.00 ± 0.18 and 1.98 ± 0.12*e*, respectively. These results show that, statistically:

(i) The number of delocalized electrons by **ad** waters in 3D hexamers examined is the same as found for the cyclic hexamer for which ^intermol^*N*^deloc^ = 1.557 ± 0.001*e* [47].
(ii) The difference in ^intermol^*N*^deloc^ between **aad** and **add** waters in 3D hexamers is insignificant.
(iii) Most importantly, there is a very significant difference in the number of delocalized electrons between **ad** and double-acceptor **aad** and double-donor **add** waters, with **ad** delocalizing about 0.45*e* less.

**Figure 5:**
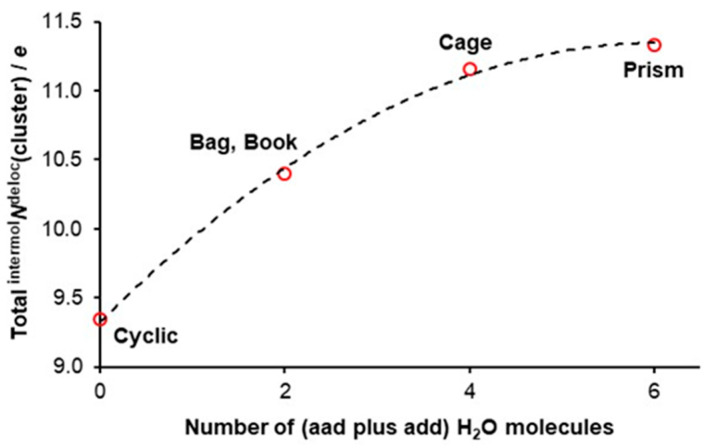
The trend in the total number of electrons intermolecularly delocalized by all water molecules in a cluster, and the number of **aad** plus **add** water molecules in a cluster. Regression for the fitted data (dashed line) is also indicated.

The data seen in Figure 5 show a non-linear, asymptotic increase in the total number delocalized by all six water molecules electrons throughout a cluster, from the smallest value found in the cyclic 2D homodromic structure where only **ad** water molecules are present to the largest value computed for prism where there are no **ad** molecules. The impact of double functionality (**aad**/**add**) is instantly noticeable, as a large increase, by over 1*e*, is observed already in the bag and book, where only two **ad** waters are replaced by a pair of **aad** and **add** molecules.

**Figure 6:**
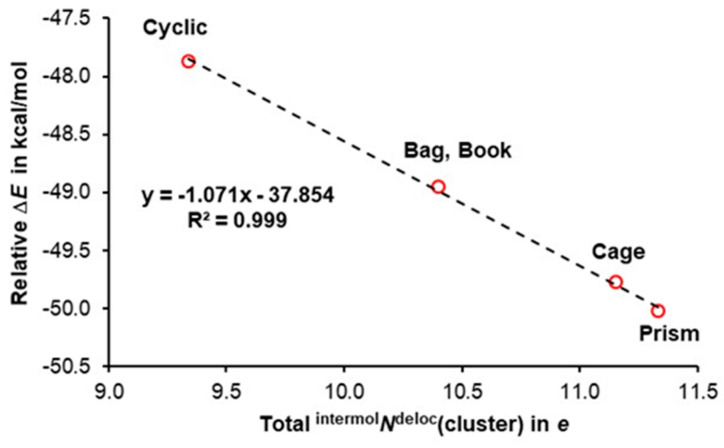
Relative to the *E* of six non-interacting water molecules, the trend in the decrease in *E*(cluster) plotted vs. the total number of electrons intermolecularly delocalized by all water molecules in the indicated hexamers. Regression for the fitted data (dashed line) is also indicated.

There is a striking ‘similarity’ between the trends seen in Figure 4 and Figure 5, where, (i) in Figure 4, an asymptotic decrease in electronic energy of hexamers with the number of water molecules having **aad** plus **add** functionality is presented, and (ii) in Figure 5, where asymptotic increase in the number of the total ^intermol^*N*^deloc^ with the number of waters having double functionality is seen. These asymptotic trends, having similar shapes but going in the opposite directions, suggest that there should be a direct link between cluster stability and the total number of delocalized electrons throughout the cluster. Indeed, an excellent linear correlation is observed in Figure 6, showing that the least stable cyclic structure has the smallest number of *e*-delocalized, and the opposite is observed for the lowest energy hexamer, prism. Clearly, **aad** and **add** waters cooperate more (delocalize more electrons) and, hence, stabilize clusters more (decrease their electronic energy more), and this is in a strong support of our two hypotheses.

We would like to conclude this section by stating that, from the MOWeD-based perspective, our findings contradict the decades-old concept of **aad** and **add** functionality being involved in the anticooperativity when electronic stabilization of entire water clusters occurs. Each water molecule with **aad** or **add** functionality is involved in three intermolecular H-bonding with other water molecules, resulting in three different ‘communication channels’ with the remaining water molecules due to unique molecular environment impacting the delocalization of electrons through these H-bridges. Each local H-bridge facilitates *e*-delocalization but to a different degree. Hence, one is faced with a serious challenge when interpretation of the role played by each local H-bridge occurs in terms of which one facilitates (cooperativity) and which one obstructs (anticooperativity) the delocalization of electrons. Clearly, a localized approach must involve an arbitrarily selected reference state versus which selected property of each H-bridge is quantified; typically, properties of a water dimer are used for that purpose. To minimize or even avoid an arbitrary approach, it is then reasonable to consider a significant increase in the total number of electrons delocalized by all water molecules throughout the space occupied by 3D clusters as a principal driver and a quantifiable measure of cooperativity, as we already stipulated for the 2D cyclic clusters [48].

Finally, one might argue that the total number of delocalized electrons does not provide an insight into individual water contributions with different functionalities. One might ask, for instance, if it is possible that, in a specific cluster, an **ad** water molecule is delocalizing more electrons than either **aad** or **add** waters believed to be involved in anticooperativity? To address this, relevant data are presented in Table 2, where the number of electrons intermolecularly delocalized by each individual water molecule to the remaining five waters is presented; for brevity, it is shown as *N*^deloc^ in Table 2, where, for convenience, the functionality of each water and its number (as in Figure 1) are also included.

The impact of an immediate environment on ^intermol^*N*^deloc^ is immediately seen in Table 2. For instance, ^intermol^*N*^deloc^ varies between 2.11 and 1.78*e* in reasonably the symmetrical structure of prism, where, in addition, there are no **ad** waters. A large spread of delocalized electrons by **ad** molecules is also seen for the book (from 1.65 to 1.41*e*) and bag (from 1.72 to 1.41*e*) structures. Fundamentally important, however, is the finding that the **aad** and **add** water molecules in the cage, book or bag delocalize significantly more electrons than **ad** molecules of these hexamers, and this is in direct contrast to the common view of these waters being involved in anticooperativity and provides a quantified support for our **hypothesis 2**.

### 3.4. Quantifying Individual Water’s Contribution to the Cluster’s Stability

Classically, delocalizing electrons between two atoms is known to be the measure of the covalent character of chemical bonding, as defined by Lewis, e.g., two electrons shared (delocalized) between two neighboring atoms represents a single covalent bond. Because, in Lewis’ interpretation of bonding, one considers shared electrons next to each other’s atoms, it implies that the process of covalent bonding is an intramolecular event. Actually, according to a classic dogma, atoms are bonded only when they are connected through covalent bonds (purely ionic interactions excluding), and in addition, atoms constituting a molecule (they are covalently bonded) might be involved in either bonding or non-bonding interactions with atoms of the same molecule (intramolecular interactions) or atoms of another molecule (intermolecular interactions).

Our all-body MOWeD-based data presented here and previously for cyclic structures [48] reveal that a large number of electrons is delocalized among water molecules in clusters (see Figure 5), showing that over 11 electrons are delocalized intermolecularly in prism, and this is about 2*e* more than we found in the cyclic hexamer. Furthermore, all the above results clearly demonstrate that gaining stability is synonymous with delocalizing electrons throughout a cluster. The intermolecular *e*-delocalization can then be seen as intermolecular bonding of a covalent character when the Lewis concept is expanded to the entire molecular system.

We have estimated [48] that the energetic effect of intermolecular *e*-delocalization on the stability of homodromic cyclic water clusters, where **ad** functionality is exclusively observed, is about an order of magnitude smaller when compared with intramolecular *e*-delocalization in a classical covalent single bond formation. Considering the theme of this contribution, it is of utmost interest and importance to quantify the energy contributions made to a molecular system’s stability by the different functionality of water molecules. To achieve that, a FAMSEC protocol dedicated for that purpose has been used. It is incorporated in the molecular-wide and electron density (MOW*e*D)-based all-body approach. We have computed a number of energy terms describing contributions made by a water molecule toward all remaining five water molecules of the cluster. As the **aad** and **add** functionalities were always linked with anticooperativity (or negative cooperativity or not strict cooperativity), data obtained for bag and cage hexamers will be discussed in some detail, as they have waters with **ad**, **aad** and **add** functionalities. Recall that bag and book hexamers have exactly the same number of **ad**, **aad** and **add** water molecules (descriptors computed for bag and book are highly comparable), whereas prism does not have **ad** water molecules.

Data pertaining to the bag hexamer are included in Table 3. The trends seen in the figures that follow were computed for the cage hexamer, as highly comparable indices were obtained for all 3D hexamers; relevant data obtained for book, cage and prism are in Table S1 in the Supplementary Materials. To ease interpretation, water molecules in Table 3 follow the order of their interaction energy with the remaining five waters, *E*_int_(W,R), from the most attractive (most negative) to the least attractive. It is immediately seen in Table 3 that water molecules with **aad** and **add** functionalities are involved in the strongest interactions with the remaining five waters in the cluster.

Remarkably, and regardless of functionality of a water molecule, intermolecular interactions are governed predominantly by the exchange correlation (quantum) component *V*_XC_(W,R) that constitutes, on average, about 71.5 ± 0.5% for 3D structures (69% for a cyclic hexamer) of the total intermolecular interaction energy, *E*_int_(W,R).

**Figure 7:**
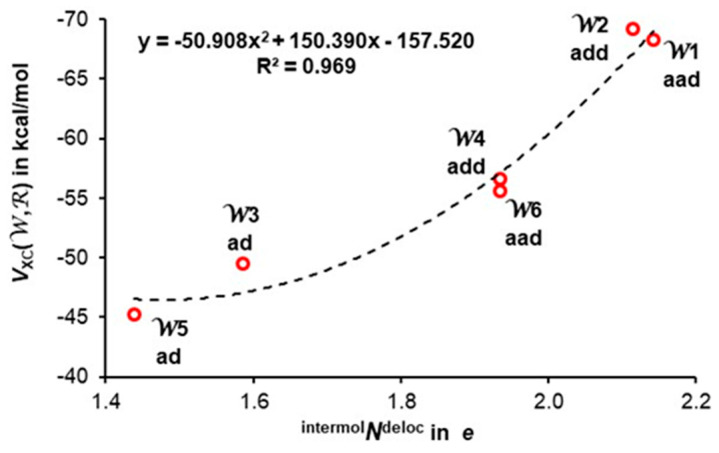
A trend between intermolecularly delocalized electrons (^intermol^*N*^deloc^) and the XC term of a total interaction energy between a water molecule and the remaining five waters *V*_XC_(W,R) computed for each individual molecule in the cage hexamer.

By combining intermolecularly delocalized electrons ^intermol^*N*^deloc^ (shown as *N*^deloc^ in Table 2) with the *V*_XC_(W,R) component of the interaction energy included in Table 3, one obtains a reasonable correlation; as an example, the data obtained for the cage hexamer is plotted in Figure 7. It shows that an increase in the number of delocalized electrons throughout a cluster is followed by a more significant contribution of the XC term (quantum component) to the interaction energy *E*_int_(W,R). Furthermore, and importantly, **ad** water molecules (i) delocalize less electrons and (ii) contribute the least to the stability of a cluster as measured by the *V*_XC_(W,R) interaction energy term, always stabilizing in nature. This finding is in direct conflict with a commonly accepted and followed for decades concept of anticooperativity attributed to **aad** and **add** functionality of molecules.

The above has demonstrated the importance of the *V*_XC_ term in understanding water functionality and its cooperativity. There are, however, other energy components impacting the (in)stability of a system. The strongest evidence regarding individual water’s contribution to a cluster’s (in)stability, i.e., (anti)cooperativity, should come from the purposely derived energy term *mol*-FAMSEC. We found highly comparable trends between ^intermol^*N*^deloc^ and *V*_XC_(W,R), as well as between ^intermol^*N*^deloc^ and *mol*-FAMSEC (see Figure S1 in the Supplementary Materials prepared for cage as an example). This is an important discovery, as it illustrates that there is a strong link between the total number of electrons delocalized by a water molecule throughout the space occupied by a 3D cluster (^intermol^*N*^deloc^) and the quantifiable measure of energy contribution to the stability of a cluster made by a water molecule (*mol*-FAMSEC). Recall that the ^intermol^*N*^deloc^ term is interpreted here as a principal driver and quantifiable measure of cooperativity. Furthermore, the data seen in Figure S1 in the Supplementary Materials suggest that there should be another important and nearly linear correlation between the quantum component of the interaction energy a water molecule is involved in with the remaining waters of a cluster, *V*_XC_(W,R) and *mol*-FAMSEC. Figure S2 in the Supplementary Materials shows that such a linear correlation indeed exists in the cage hexamer. Actually, we have established that high-quality linear correlation between *V*_XC_(W,R) and *mol*-FAMSEC exists for all 3D hexamers discussed in this work. In each case, the most significant *mol-*FAMSEC term is observed for water molecules with **aad** and **add** functionality in a specific cluster, and this strongly suggests that they are not involved in anticooperativity.

**Figure 8:**
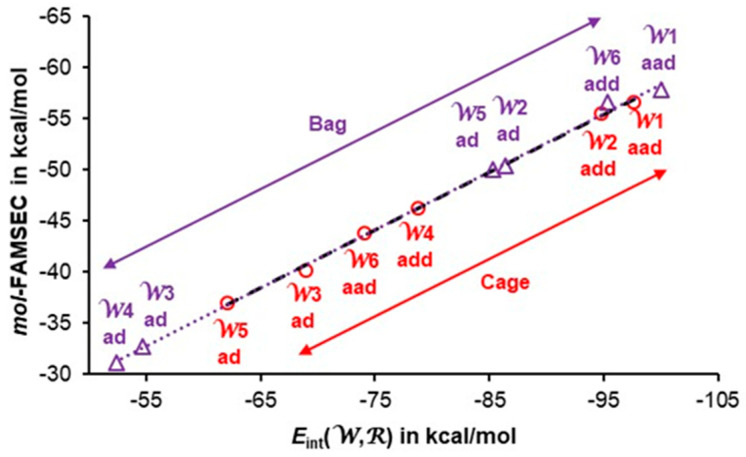
A correlation between the interaction energy *E*_int_(W,R) and the *mol*-FAMSEC energy terms computed for each individual molecule in the cage hexamer (red color) and bag hexamer (violet color). Black dashed and dotted violet lines are the trend lines for the cage and bag, respectively.

The data presented in Figure 8 show a nearly perfect correlation between *mol*-FAMSEC and the interaction energy *E*_int_(W,R) computed for the cage (red circles) and bag (violet triangles) hexamers. The term *E*_int_(W,R) stands for the interaction energy between the water molecule W*n* and remaining five water molecules R in a cluster indicated in Figure 8. Similar trends are also observed for the remaining 3D water hexamers discussed in this work. This means that the combined data from all 3D hexamers follow a single linear trend that might be used as a predictive tool of either *mol*-FAMSEC or *E*_int_(W,R) (see Figure S3 in the Supplementary Materials).

To fully understand the trend seen in Figure 8, it is fundamentally important to link it with the trends and expressions discussed above. According to our definition of cooperativity, it is the physics- and quantum-based quantifiable intermolecular electron delocalization that drives the formation of water clusters (clusters of molecules in general), and it leads to the non-additive decrease in their electronic energy on a *n*-membered cluster formation. The data in Figure 7 show a good non-linear correlation between the driving force of cooperativity, i.e., the number of intermolecularly delocalized electrons, and quantum contribution to the interaction energy *V*_XC_(W,R). In the IQA-defined energy partitioning scheme, the interaction energy between any pair of atoms, A and B, is made from two principle components, namely the classic Coulomb term *V*_cl_(A,B) and the XC term (commonly interpreted as covalent or quantum contribution) *V*_XC_(A,B). As stated above, the contribution of the *V*_XC_ term to the interaction energy is nearly constant (about 71%), meaning that the %-fraction of the classic Coulomb contribution to the interaction energy made by individual water molecules is also constant and much smaller. According to classical orbital-based interpretations, the intermolecular interactions, such as, e.g., intermolecular H-bonds, are dominated by a classical term. One must stress here that the individual diatomic classic Coulomb intermolecular interaction energy terms *V*_cl_(A,B) in water clusters vary between large positive (repulsive) and large negative (attractive) values. However, their opposite in sign contributions largely cancel each other out, whereas always negative *V*_XC_(A,B) terms sum up to a significant total value of *V*_XC_(W,R).

As shown in Section 2.2., the *mol*-FAMSEC term is made up of two major contributions, namely *loc*-FAMSEC and the interaction energy of a selected fragment with all the remaining atoms of a molecular system, which is equivalent, in our case, to the *E*_int_(W,R) term. From this and the relationship seen in Figure 8, it follows that the *loc*-FAMSEC term contributes a nearly constant %-fraction to the *mol*-FAMSEC energy term computed for individual water molecules in all 3D water clusters. This fully explains the linear relationship observed in Figure 8 and Figure S2 in the Supplementary Materials.

The above undisputedly illustrates that this is indeed not the **ad** mode of action of water molecules but rather **aad** and **add** that stabilize a cluster more. Actually, we have shown that, regardless of the physical/quantum property or energy component considered, regardless of the number of **ad**, **aad** and **add** waters in each cluster, the **aad** and **add** functionality always provides the most significant contribution within a specific cluster. This does not mean, however, that **ad** waters always provide smaller contributions relative to that made by **aad** and **add** molecules. This is clearly seen in Figure 8, where waters W2 and W5 (both **ad**) of the bag hexamer provide more significant contribution to the bag’s stability than energy contributions made to the cage by its waters W4-**add** and W6-**aad**. This nicely illustrates the overall complexity of cluster formation and the impact made by immediate, as well distant, neighbors, the relative orientation of water molecules, distances between O-atoms and many more cooperativity-induced effects, such as *V*_XC_(W,R), *E*_int_(W,R) and *mol*-FAMSEC energy terms, on the role played by a single water molecule.

### 3.5. Quantifying Individual Intermolecular H-Bond Contributions to the Cluster’s Stability

Considering all the above, it should then be easy to comprehend that three-way interacting **aad** and **add** molecules will show a significant spread in the computed descriptors of any kind along each H-bonded link. From the all-body MOWeD approach, we conclude that the differences in values observed for the computed indices along each H-bond have nothing to do with anticooperativity, as universal and quantifiable quantum and physics-based processes apply throughout all the 3D space occupied by the cluster. *Clearly, local environment determines, to some degree, the effectiveness of cooperativity between all water molecules along all possible H-bonded links that provide a unique, ‘privileged’ and most effective mode of transport for electrons delocalized predominantly by O-atoms throughout a molecular system, as documented previously for cyclic structures* [48] *and here for 3D hexamers discussed* (an example for the bag is shown in Figure S4 in the Supplementary Materials).

Notwithstanding the above, and since it has been accepted for decades that hydrogen bonds may mutually either enhance their strength when they are involved in cooperativity or reduce their strengths when involved in anticooperativity, we decided to analyze the intermolecular H-bonds in all the clusters examined. We decided to make use of energy contributions made to a cluster by H-bonds using the *mol*-FAMSEC energy term dedicated for this purpose. One must recall that, the stronger a H-bond is, the more significant the energy-stabilizing contribution to a molecular system it provides. When making use of a classical approach that is focused on individual H-bonds, it is apparent that the values of *mol*-FAMSEC seen in Table 4 do not follow an obvious pattern that might be used to support the concept of anticooperativity.

A large spread in values of the *mol*-FAMSEC term is seen in Table 4, e.g., between about –110 and –168 kcal/mol in the book. As one would expect, most values computed for the more stable 3D clusters are more significant than that of –115 kcal/mol obtained for a homodromic cyclic hexamer. However, the *mol*-FAMSEC terms obtained for HB-7 in the cage and book are less significant.

Focusing on three H-bonds of the most comparable W1 water molecule in all 3D clusters (it has the same **aad** functionality throughout), they show different distributions of *mol*-FAMSEC values in each cluster. This is due to, in our view, different overall molecular environments, and we see in Table 4 that *mol-*FAMSEC: (i) gradually becomes more negative in prism and cage, (ii) has a much more negative value for HB-2 in the book hexamer, whereas comparable and less significant *mol*-FAMSEC terms are seen for HB-1 and HB-3 and (iii) highly comparable values for all three H-bonds we found in the bag cluster. Variation in the *mol*-FAMSEC values seen in Table 4 for W1 water cannot be linked with anticooperativity, and any selection made would be highly arbitrary and would have to be supported.

**Figure 9:**
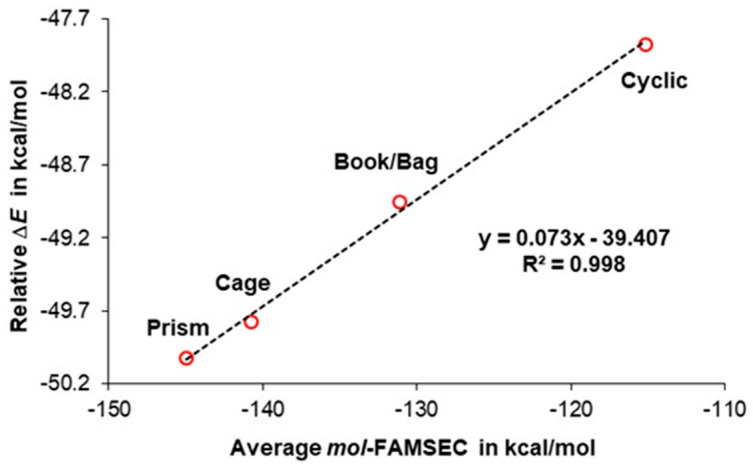
A linear trend obtained between average *mol*-FAMSEC that was computed for intermolecular H-bonds OH···O formed in each hexamer and, relative to the non-interacting six water molecules, an increase in the hexamer’s stability.

A very different and consistent picture emerged when the impact of the molecular environment was minimized by making use of the averaged values of the *mol*-FAMSEC terms seen in Table 4. Based on chemists’ accumulated experience and knowledge, the larger the number of intermolecular hydrogen bonds, the more significant the contribution to the water cluster’s stability is expected, and this is exactly what one can see in Figure 9. Relative to the non-interacting six water molecules, prism is the most stable, and it forms the largest number (nine) of intermolecular hydrogen bonds. Furthermore, and importantly, on average, the stabilizing contribution made by a H-bond (as measured by the *mol*-FAMSEC term) increases from a cyclic to prism structure.

Finally, recall that, according to our understanding and definition of the cooperativity phenomenon, it is synonymous with the intermolecular **all-body** *e*-delocalization leading to the increase in stability of water molecules on a cluster formation. Analysis of the data in Figure 9 leads to another supporting observation, namely the largest number of intermolecularly delocalized electrons found for prism (see Figure 6) can be attributed to the largest number of density bridges (DBs) linking water molecules due to the formation of classical intermolecular H-bonds. It then becomes clear that it is not a single DB but a network of DBs that facilitates the most efficient *e*-delocalization that, in turn, determines the stability of a cluster.

## 4. Computational Methods

All calculations were performed in Gaussian 09 Rev. D.01 [69] in the gas phase with a keyword ‘opt = verytight’ at the B3LYP level of theory with Grimme’s [70] empirical correction for dispersion using the keyword ‘empiricaldispersion = GD3′. The Dunning triple zeta basis set, aug-cc-pVTZ, which is augmented by diffuse functions, was used throughout. Coordinates of all optimized structures are given in the Supplementary Materials. Frequency calculations were performed for the optimized structures to verify that no imaginary frequency was present. Topological, QTAIM [58] molecular graphs, atomic overlap matrices and IQA [62,63] calculations were performed in AIMAll [71] using B3LYP-generated wavefunctions. The IQA energy terms, and interaction energies in particular, were found to be highly comparable to those obtained at the CCSD/BBC1 level [72]. FAMSEC and FALDI data were calculated using in-house software, and FALDI isosurfaces were visualized using VMD ver. 7 [73]. FALDI codes were incorporated in MOW*e*D-LAC (molecular-wide electron delocalization and localization atomic counts) and MOW*e*D-LFC (molecular-wide electron delocalization and localization fragment counts) applications; these two applications are made freely available [74].

## 5. Conclusions

The proposed here general definition of the cooperativity phenomenon is a straightforward extension of our definition proposed for homodromic cyclic clusters of water [48]. The expanded definition should be applicable to any molecular cluster, as it states that ‘*the quantifiable, classical physics- and quantum-based cooperativity phenomenon is synonymous with the intermolecular **all-body** delocalization of electrons, leading to the increase in stability of individual molecules on an n-membered cluster formation.* From this, it follows that a non-additive decrease in the electronic energy of clusters, from the least stable homodromic cyclic hexamer to most stable prism, is a direct result of a non-linear increase in the total number of intermolecularly delocalized electrons throughout a 3D space occupied by a hexamer, ^intermol^*N*^deloc^. Water molecules in 3D hexamers studied in this work can exhibit three functionalities, namely they can act as a proton acceptor and proton donor (**ad**), a double proton acceptor and a proton donor (**aad**) and a single proton acceptor and a double proton donor (**add**). Waters with a double functionality (i.e., **aad** and **add**) are typically associated with anticooperativity (or negative cooperativity or not strict cooperativity). Remarkably, prism is the most stable hexamer among all possible 3D configurations of the six water molecules (about 80) and has all six water molecules involved in anticooperativity if one accepts the decades-long interpretation. Therefore, how is it possible that prism is the most stable among them all? Our definition of cooperativity fully explains these two contradicting observations. We showed that all **aad** and **add** waters outperform **ad** molecules when the number of intermolecularly delocalized electrons is accounted for. This means that, in all the 3D hexamers studied, individual ‘unticooperators’, **aad** and **add** water molecules, delocalized significantly more electrons than always ‘cooperating’ **ad** waters. Hence, one is left with only one possible conclusion, that the **aad** and **add** water molecules in 3D hexamers are the best ‘cooperators’, and this excludes them as being involved in anticooperativity. It would be of interest and paramount importance to establish if our finding is equally applicable to all remaining 70+ 3D hexamers. This would be a mammoth job, but due to its fundamental importance, we intend to embark on that challenge and hope to report our results soon.

In our opinion, the ^intermol^*N*^deloc^ term is a universal quantifiable measure of cooperativity phenomenon. It can be applied to the entire cluster (then it stands for the total number of electrons delocalized by all molecules of a cluster) or individual molecules within a cluster (both these options were explored in this work) or even to examine cooperativity/anticooperativity of a *k*-membered molecular fragment within a *n*-membered cluster (*k* < *n*).

One must stress that the physics- and quantum-based processes of *e*-delocalization lead to changes in properties of all imaginable properties of a molecular system, such as a water cluster. It is apparent that some cooperativity-induced changes in the local properties of a cluster were interpreted as anticooperativity. We investigated a few cooperativity-induced properties, but from the molecular-wide and electron density (MOWeD) perspective, we established that the changes in these properties do not corroborate with the commonly accepted concept of anticooperativity. Our main focus was on contributions made to cooperativity-induced properties by individual water molecules within the 3D clusters examined. Contrary to common interpretations, the **aad** and **add** molecules have not shown any signature of anticooperativity, as they always contributed the most to cluster stability, regardless of the descriptor investigated. Actually, we discovered an excellent linear relationship between *mol*-FAMSEC (a dedicated energy term to quantify the energy contribution made by a selected molecular fragment to the stability of a molecular system) and the intermolecular interaction energy a fragment (here, a water molecule) is involved in with the remaining atoms of a system (here, a cluster). Remarkably, within each hexamer investigated, all water molecules with the **aad** and **add** functionalities outperformed the classically accepted ‘cooperators’, i.e., the **ad** water molecules, by stabilizing a cluster the most due to most significant *mol*-FAMSEC.

## Figures and Tables

**Table 1:** Comparison of the relative energies, as Δ*E* = *E*(hexamer) − *E*(prism) values in kcal/mol, obtained at different levels of theory for the indicated water hexamers.

		Δ*E* = *E*(hexamer) − *E*(prism) in kcal/mol				
Source	Level of Theory	Cyclic	Bag	Book	Cage	Prism
Our data	B3LYP/aug-cc-pVTZ/GD3	2.15	1.34	0.80	0.25	0
Bates et al. [64]	CCSD(T)/CBS	2.48	1.62	0.87	0.25	0
Kryachko [65]	MP2(full)/aug-cc-pVDZ	2.06	N/A	1.16	0.25	0
Olson et al. [66]	CCSD(T)/aug-cc-pVTZ	2.10	N/A	1.20	0.30	0

**Table 2:** The number of electrons delocalized by an indicated water molecule to the remaining five water molecules of the 3D hexamers. The functionality of the water molecules and their numbering is consistent with that seen in Figure 1.

Prism		Cage		Book		Bag	
Water	*N* ^deloc^	Water	*N* ^deloc^	Water	*N* ^deloc^	Water	*N* ^deloc^
**aad** W1	2.112	**aad** W1	2.142	**aad** W1	2.100	**aad** W1	2.188
**add** W2	2.027	**add** W2	2.116	**add** W4	2.048	**add** W6	2.074
**aad** W3	1.771	**add** W4	1.935	**ad** W2	1.647	**ad** W2	1.719
**add** W4	1.861	**aad** W6	1.935	**ad** W3	1.484	**ad** W3	1.433
**aad** W5	1.778	**ad** W3	1.587	**ad** W5	1.603	**ad** W4	1.411
**add** W6	1.786	**ad** W5	1.439	**ad** W6	1.412	**ad** W5	1.677

**Table 3:** Energy terms computed for individual water molecules in the bag hexamer. *E*_int_(W,R) and *V*_XC_(W,R) stand for the interaction energy and its exchange correlation (covalent or quantum) component, respectively, computed between an indicated water molecule and the remaining waters. *mol*-FAMSEC quantifies the energy contribution made by a water molecule to the stability of the bag hexamer.

	Energy Terms in kcal/mol		
Water	*E*_int_(W,R)	*V*_XC_(W,R)	*mol*-FAMSEC
**aad** W1	−100.03	−70.22	−57.81
**add** W6	−95.36	−69.20	−56.58
**ad** W2	−86.34	−59.52	−50.39
**ad** W5	−85.31	−59.23	−50.07
**ad** W3	−54.68	−40.56	−32.69
**ad** W4	−52.42	−39.20	−31.12
Average:	−79.0	−56.3	−46.4
St. Dev.:	20.5	13.6	11.7

**Table 4:** *mol*-FAMSEC-quantified contributions to the cluster’s stability made by the indicated classical intermolecular H-bonds.

	Water cluster				
	Prism	Cage	Book	Bag	Cyclic
H-Bond	*mol*-FAMSEC in kcal/mol				
HB-1	–141.0	–139.3	–139.5	–142.1	–115.2
HB-2	–157.3	–145.1	–167.7	–142.7	
HB-3	–163.0	–161.0	–143.4	–143.7	
HB-4	–126.4	–133.0	–119.4	–121.8	
HB-5	–156.9	–161.5	–118.8	–119.2	
HB-6	–139.1	–150.1	–119.4	–122.4	
HB-7	–124.4	–109.8	–109.2	–125.5	–
HB-8	–138.1	–126.3	–	–	–
HB-9	–158.5	–	–	–	–
Average*:*	–145.0	–140.8	–131.1	–131.1	–115.2

## Data Availability

On request, computational data are available from I.C.

## Supplementary Materials

The following supporting information can be downloaded at https://www.mdpi.com/article/10.3390/molecules30091944/s1: The Supporting Information is available free of charge as Cartesian XYZ coordinates of 3D water clusters; diatomic distances between O atoms and H and O atoms in 3D hexamers; energies of 3D clusters; the total interaction energy and its exchange–correlation term between a water molecule and remaining waters in clusters and mol-FAMSEC energy term computed for each water molecule in book, cage and prism; Figures showing trends between selected descriptors computed for individual water molecules in clusters and visual presentation of FALDI 3D density distributions.
