# Supplementary material for: The Common Concept of Anticooperativity Among Molecules Is Fundamentally Flawed, Based on Novel and Unified Molecular-Wide and Electron Density (MOWeD) Concept of Chemical Bonding
Source: Molecules. 2025 Apr 27;30(9):1944. doi: 10.3390/molecules30091944 (PMC12073546; doi:10.3390/molecules30091944)
Supplement: Supplementary file 1 [file molecules-30-01944-s001.zip › molecules-3472201-supplementary.pdf]

The Common Concept of Anticooperativity Among Molecules is Fundamentally Flawed, Based on Novel and Unified Molecular-Wide and Electron Density (MOWeD) Concept of Chemical Bonding

Supplementary Materials

Cartesian coordinates of optimized 3D hexamers

Bag hexamer

Atom No.	Symbol	X	Y	Z
1	O	-0.946682	2.398653	-1.633665
2	H	-0.071422	2.113247	-2.012300
3	H	-1.375495	2.939249	-2.302203
4	O	1.403848	1.513875	-2.518945
5	H	1.714486	0.855100	-1.846566
6	H	2.116150	2.154593	-2.597305
7	O	2.152934	-0.118601	-0.539735
8	H	1.470874	-0.013553	0.175108
9	H	2.347404	-1.056562	-0.603831
10	O	0.214191	0.313167	1.254613
11	H	0.167880	1.280495	1.365239
12	H	-0.623089	0.096176	0.805933
13	O	-0.208297	3.113187	1.004603
14	H	-0.541424	3.023106	0.092988
15	H	-0.933971	3.479524	1.517385
16	O	-2.110735	0.183763	-0.371031
17	H	-1.802633	0.920521	-0.932358
18	H	-2.312318	-0.541640	-0.967831

Zero-point correction= 0.148442 (Hartree/Particle)
Thermal correction to Energy= 0.163416
Thermal correction to Enthalpy= 0.164360
Thermal correction to Gibbs Free Energy= 0.107296
Sum of electronic and zero-point Energies= -458.726372
Sum of electronic and thermal Energies= -458.711398
Sum of electronic and thermal Enthalpies= -458.710454
Sum of electronic and thermal Free Energies= -458.767518

Interatomic distances between O-atoms in the bag hexamer.

Atom A	Atom B	d(A,B) / Å
O1	O4	2.66300
O1	O7	4.14015
O1	O10	3.74687
O1	O13	2.83129
O1	O16	2.80268
O4	O1	2.66300
O4	O7	2.67271
O4	O10	4.13482
O4	O13	4.19192
O4	O16	4.32840
O7	O1	4.14015
O7	O4	2.67271
O7	O10	2.67672
O7	O13	4.29009
O7	O16	4.27770
O10	O1	3.74687
O10	O4	4.13482
O10	O7	2.67672
O10	O13	2.84273
O10	O16	2.83985
O13	O1	2.83129
O13	O4	4.19192
O13	O7	4.29009
O13	O10	2.84273
O13	O16	3.75409
O16	O1	2.80268
O16	O4	4.32840
O16	O7	4.27770
O16	O10	2.83985
O16	O13	3.75409

Interatomic distances between H- and O-atoms in the bag hexamer

Atom A	Atom B	d(A,B) / Å	Atom A	Atom B	d(A,B) / Å
H2	O4	1.67103	H11	O1	3.38909
H2	O7	3.47812	H11	O4	4.08276
H2	O10	3.74093	H11	O7	3.08656
H2	O13	3.18124	H11	O13	1.90534
H2	O16	3.25199	H11	O16	3.06750
H3	O4	3.13104	H12	O1	3.37013
H3	O7	4.99065	H12	O4	4.14405
H3	O10	4.69833	H12	O7	3.09245
H3	O13	3.51106	H12	O13	3.05186
H3	O16	3.44423	H12	O16	1.89895
H5	O1	3.08378	H14	O1	1.88029
H5	O7	1.68764	H14	O4	3.58944
H5	O10	3.48739	H14	O7	4.18691
H5	O13	4.11403	H14	O10	3.04370
H5	O16	4.15454	H14	O16	3.27718
H6	O1	3.22011	H15	O1	3.33130
H6	O7	3.06633	H15	O4	5.06173
H6	O10	4.67392	H15	O7	5.16790
H6	O13	4.39269	H15	O10	3.37833
H6	O16	5.16788	H15	O16	3.97655
H8	O1	3.86458	H17	O1	1.84644
H8	O4	3.09765	H17	O4	3.62641
H8	O10	1.68859	H17	O7	4.10858
H8	O13	3.64475	H17	O10	3.03633
H8	O16	3.62838	H17	O13	3.33189
H9	O1	4.88365	H18	O1	3.30963
H9	O4	3.34142	H18	O4	4.52117
H9	O10	3.14334	H18	O7	4.50563
H9	O13	5.14835	H18	O10	3.47177
H9	O16	4.63331	H18	O13	4.65566

Book hexamer

Atom No.	Symbol	X	Y	Z
1	O	-0.329771	3.565719	0.499029
2	H	0.532944	3.628064	0.014979
3	H	-0.294882	4.207850	1.212900
4	O	2.035411	3.480853	-0.757440
5	H	2.229693	2.510308	-0.742131
6	H	2.134632	3.759553	-1.671518
7	O	2.240415	0.806028	-0.622952
8	H	1.385127	0.622915	-0.157462
9	H	2.923408	0.353005	-0.121889
10	O	-0.100007	0.654917	0.689887
11	H	-0.251561	1.598980	0.850731
12	H	-0.889848	0.362202	0.192297
13	O	-2.434678	0.246002	-0.788849
14	H	-2.624156	1.200964	-0.899587
15	H	-3.206188	-0.128874	-0.355781
16	O	-2.652815	3.001693	-0.901500
17	H	-1.841372	3.303071	-0.444458
18	H	-2.673451	3.460949	-1.745055

Zero-point correction= 0.148658 (Hartree/Particle)
Thermal correction to Energy= 0.163576
Thermal correction to Enthalpy= 0.164520
Thermal correction to Gibbs Free Energy= 0.107132
Sum of electronic and zero-point Energies= -458.727027
Sum of electronic and thermal Energies= -458.712109
Sum of electronic and thermal Enthalpies= -458.711165
Sum of electronic and thermal Free Energies= -458.768553

Interatomic distances between O-atoms in the book hexamer.

Atom A	Atom B	d(A,B) / Å
O1	O4	2.67955
O1	O7	3.93454
O1	O10	2.92609
O1	O13	4.13640
O1	O16	2.77058
O4	O1	2.67955
O4	O7	2.68604
O4	O10	3.82631
O4	O13	5.51787
O4	O16	4.71485
O7	O1	3.93454
O7	O4	2.68604
O7	O10	2.68774
O7	O13	4.71144
O7	O16	5.37050
O10	O1	2.92609
O10	O4	3.82631
O10	O7	2.68774
O10	O13	2.79366
O10	O16	3.81532
O13	O1	4.13640
O13	O4	5.51787
O13	O7	4.71144
O13	O10	2.79366
O13	O16	2.76661
O16	O1	2.77058
O16	O4	4.71485
O16	O7	5.37050
O16	O10	3.81532
O16	O13	2.76661

Interatomic distances between H- and O-atoms in the book hexamer

Atom A	Atom B	d(A,B) / Å	Atom A	Atom B	d(A,B) / Å
H2	O4	1.69579	H11	O1	1.99947
H2	O7	3.35951	H11	O4	3.37015
H2	O10	3.11380	H11	O7	3.00174
H2	O13	4.57070	H11	O13	3.04709
H2	O16	3.37362	H11	O16	3.28693
H3	O4	3.13704	H12	O1	3.26654
H3	O7	4.62282	H12	O4	4.38008
H3	O10	3.59651	H12	O7	3.26499
H3	O13	4.92767	H12	O13	1.83375
H3	O16	3.38900	H12	O16	3.35728
H5	O1	3.03401	H14	O1	3.57944
H5	O7	1.70848	H14	O4	5.18938
H5	O10	3.30464	H14	O7	4.88841
H5	O13	5.18513	H14	O10	3.03248
H5	O16	4.90976	H14	O16	1.80096
H6	O1	3.28970	H15	O1	4.75967
H6	O7	3.13592	H15	O4	6.37698
H6	O10	4.49540	H15	O7	5.53271
H6	O13	5.83119	H15	O10	3.36988
H6	O16	4.90784	H15	O16	3.22560
H8	O1	3.46871	H17	O1	1.80114
H8	O4	2.99176	H17	O4	3.89346
H8	O10	1.71016	H17	O7	4.78833
H8	O13	3.88994	H17	O10	3.36628
H8	O16	4.74522	H17	O13	3.13310
H9	O1	4.61414	H18	O1	3.24649
H9	O4	3.31299	H18	O4	4.81136
H9	O10	3.14502	H18	O7	5.69682
H9	O13	5.40050	H18	O10	4.51944
H9	O16	6.22235	H18	O13	3.36262

Cage hexamer

Atom No.	Symbol	X	Y	Z
1	O	-1.946305	3.400566	-0.130214
2	H	-1.361061	2.656298	0.183390
3	H	-2.203126	3.904851	0.645900
4	O	-0.317772	1.343904	0.324705
5	H	-0.464147	0.917141	-0.534866
6	H	0.574849	1.736918	0.254909
7	O	-1.030632	0.889848	-2.445811
8	H	-1.969725	1.183708	-2.404844
9	H	-0.994692	0.138862	-3.043457
10	O	-3.442436	2.052384	-1.989967
11	H	-3.082933	2.621024	-1.275596
12	H	-3.810804	2.649824	-2.646653
13	O	1.914777	2.884791	-0.282678
14	H	1.429065	3.216330	-1.068945
15	H	2.804892	2.673433	-0.574304
16	O	0.095310	3.562716	-2.244628
17	H	-0.600986	3.831265	-1.625736
18	H	-0.180073	2.676437	-2.530706

Zero-point correction= 0.149202 (Hartree/Particle)
Thermal correction to Energy= 0.163816
Thermal correction to Enthalpy= 0.164760
Thermal correction to Gibbs Free Energy= 0.109541
Sum of electronic and zero-point Energies= -458.727361
Sum of electronic and thermal Energies= -458.712746
Sum of electronic and thermal Enthalpies= -458.711802
Sum of electronic and thermal Free Energies= -458.767021

Interatomic distances between O-atoms in the cage hexamer.

Atom A	Atom B	d(A,B) / Å
O1	O4	2.66250
O1	O7	3.53612
O1	O10	2.74129
O1	O13	3.89836
O1	O16	2.94368
O4	O1	2.66250
O4	O7	2.89657
O4	O10	3.95262
O4	O13	2.77984
O4	O16	3.41983
O7	O1	3.53612
O7	O4	2.89657
O7	O10	2.71589
O7	O13	4.16346
O7	O16	2.90731
O10	O1	2.74129
O10	O4	3.95262
O10	O7	2.71589
O10	O13	5.68397
O10	O16	3.85507
O13	O1	3.89836
O13	O4	2.77984
O13	O7	4.16346
O13	O10	5.68397
O13	O16	2.76031
O16	O1	2.94368
O16	O4	3.41983
O16	O7	2.90731
O16	O10	3.85507
O16	O13	2.76031

Interatomic distances between H- and O-atoms in the cage hexamer

Atom A	Atom B	d(A,B) / Å	Atom A	Atom B	d(A,B) / Å
H2	O4	1.68250	H11	O1	1.79207
H2	O7	3.18469	H11	O4	3.44066
H2	O10	3.06925	H11	O7	2.92888
H2	O13	3.31671	H11	O13	5.10221
H2	O16	2.97286	H11	O16	3.45355
H3	O4	3.19628	H12	O1	3.22063
H3	O7	4.47478	H12	O4	4.76819
H3	O10	3.45186	H12	O7	3.29655
H3	O13	4.34280	H12	O13	6.19886
H3	O16	3.70878	H12	O16	4.03147
H5	O1	2.92026	H14	O1	3.50832
H5	O7	1.99333	H14	O4	2.91542
H5	O10	3.50376	H14	O7	3.65491
H5	O13	3.09750	H14	O10	5.09260
H5	O16	3.19927	H14	O16	1.81139
H6	O1	3.04504	H15	O1	4.82699
H6	O7	3.25407	H15	O4	3.51097
H6	O10	4.61276	H15	O7	4.62547
H6	O13	1.84446	H15	O10	6.43575
H6	O16	3.13228	H15	O16	3.30494
H8	O1	3.17631	H17	O1	2.05718
H8	O4	3.19453	H17	O4	3.17355
H8	O10	1.75943	H17	O7	3.08368
H8	O13	4.74201	H17	O10	3.37208
H8	O16	3.15432	H17	O13	3.00478
H9	O1	4.47563	H18	O1	3.06697
H9	O4	3.64072	H18	O4	3.15404
H9	O10	3.28068	H18	O7	1.98054
H9	O13	4.86077	H18	O10	3.36524
H9	O16	3.68090	H18	O13	3.07984

Prism hexamer

Atom No.	Symbol	X	Y	Z
1	O	-2.092508	3.004348	0.156102
2	H	-1.107512	3.165797	0.128323
3	H	-2.526244	3.857496	0.077993
4	O	0.555728	3.104790	0.138653
5	H	0.722106	2.504224	0.895744
6	H	0.812577	2.548991	-0.612116
7	O	0.724316	0.848383	1.764207
8	H	-0.219768	0.703349	1.940106
9	H	0.888584	0.409887	0.915724
10	O	-2.171595	0.820874	1.846170
11	H	-2.294018	1.704739	1.445791
12	H	-2.723445	0.788153	2.631862
13	O	0.745880	0.497476	-1.148004
14	H	-0.228447	0.388227	-1.235030
15	H	1.147275	-0.039367	-1.835906
16	O	-1.990350	0.430346	-1.080543
17	H	-2.189232	1.371346	-0.940551
18	H	-2.208547	0.035642	-0.225626

Zero-point correction= 0.149365 (Hartree/Particle)
Thermal correction to Energy= 0.164045
Thermal correction to Enthalpy= 0.164989
Thermal correction to Gibbs Free Energy= 0.109734
Sum of electronic and zero-point Energies= -458.727591
Sum of electronic and thermal Energies= -458.712911
Sum of electronic and thermal Enthalpies= -458.711967
Sum of electronic and thermal Free Energies= -458.767221

Interatomic distances between O-atoms in the prism hexamer.

Atom A	Atom B	d(A,B) / Å
O1	O4	2.65020
O1	O7	3.89470
O1	O10	2.76227
O1	O13	4.00519
O1	O16	2.85748
O4	O1	2.65020
O4	O7	2.78608
O4	O10	3.94591
O4	O13	2.91371
O4	O16	3.88865
O7	O1	3.89470
O7	O4	2.78608
O7	O10	2.89720
O7	O13	2.93336
O7	O16	3.95434
O10	O1	2.76227
O10	O4	3.94591
O10	O7	2.89720
O10	O13	4.19301
O10	O16	2.95821
O13	O1	4.00519
O13	O4	2.91371
O13	O7	2.93336
O13	O10	4.19301
O13	O16	2.73788
O16	O1	2.85748
O16	O4	3.88865
O16	O7	3.95434
O16	O10	2.95821
O16	O13	2.73788

Interatomic distances between H- and O-atoms in the prism hexamer

Atom A	Atom B	d(A,B) / Å	Atom A	Atom B	d(A,B) / Å
H2	O4	1.66439	H11	O1	1.84198
H2	O7	3.37670	H11	O4	3.43363
H2	O10	3.09547	H11	O7	3.15358
H2	O13	3.49056	H11	O13	4.17447
H2	O16	3.11825	H11	O16	2.84581
H3	O4	3.17314	H12	O1	3.38216
H3	O7	4.73964	H12	O4	4.72608
H3	O10	3.53176	H12	O7	3.55577
H3	O13	4.84764	H12	O13	5.13888
H3	O16	3.65715	H12	O16	3.80097
H5	O1	2.95284	H14	O1	3.50058
H5	O7	1.86977	H14	O4	3.14351
H5	O10	3.48001	H14	O7	3.18040
H5	O13	2.86435	H14	O10	3.66835
H5	O16	3.94514	H14	O16	1.76916
H6	O1	3.03925	H15	O1	4.87119
H6	O7	2.92349	H15	O4	3.75959
H6	O10	4.23495	H15	O7	3.73200
H6	O13	2.12140	H15	O10	5.03116
H6	O16	3.54464	H15	O16	3.26127
H8	O1	3.46185	H17	O1	1.96944
H8	O4	3.10057	H17	O4	3.42116
H8	O10	1.95762	H17	O7	4.00973
H8	O13	3.24211	H17	O10	2.84062
H8	O16	3.51195	H17	O13	3.06946
H9	O1	4.02432	H18	O1	2.99540
H9	O4	2.82438	H18	O4	4.14651
H9	O10	3.22480	H18	O7	3.63616
H9	O13	2.07051	H18	O10	2.21592
H9	O16	3.50339	H18	O13	3.12933

Table S1. Energy terms computed for individual water molecules in the specified 3D hexamers. $E_{\text{int}}(\mathcal{W},\text{R})$ and $V_{\text{XC}}(\mathcal{W},\text{R})$ stand for the interaction energy and its exchange-correlation (covalent or quantum) component, respectively, computed between an indicated water molecule and remaining waters. *mol*-FAMSEC quantifies energy contribution made by a water molecule to the stability of a hexamer.

Data for book hexamer

	E-terms for $\mathcal{W}n$ with remaining water molecules $\mathcal{R}$ in kcal/mol			
**Water**	$E_{\text{int}}(\mathcal{W}n,\mathcal{R})$	**mol-FAMSEC**	$V_{\text{XC}}(\mathcal{W}n,\mathcal{R})$	**% of** E_{int}
aad $\mathcal{W}1$	-94.74	-55.00	-66.16	69.83
add $\mathcal{W}4$	-91.86	-54.98	-66.86	72.78
ad $\mathcal{W}2$	-82.12	-48.13	-57.07	69.50
ad $\mathcal{W}5$	-81.52	-47.84	-56.77	69.64
ad $\mathcal{W}3$	-63.67	-37.82	-45.51	71.49
ad $\mathcal{W}6$	-61.98	-36.80	-45.03	72.66
Average:	-79.3	-46.8	-56.2	71.0
St. Dev.:	13.8	8.0	9.5	1.5

Data for cage hexamer

	E-terms for $\mathcal{W}n$ with remaining water molecules $\mathcal{R}$ in kcal/mol			
**Water**	$E_{\text{int}}(\mathcal{W}n,\mathcal{R})$	**mol-FAMSEC**	$V_{\text{XC}}(\mathcal{W}n,\mathcal{R})$	**% of** E_{int}
aad $\mathcal{W}1$	-97.63	-56.69	-68.18	69.84
add $\mathcal{W}2$	-94.82	-55.54	-69.14	72.92
add $\mathcal{W}4$	-74.09	-43.82	-55.51	74.93
aad $\mathcal{W}6$	-78.75	-46.28	-56.53	71.78
ad $\mathcal{W}3$	-68.95	-40.19	-49.43	71.69
ad $\mathcal{W}5$	-62.08	-36.99	-45.24	72.87
Average:	-79.4	-46.6	-57.3	72.3
St. Dev.:	14.2	8.0	9.7	1.7

Data for prism hexamer

	E-terms for $\mathcal{W}n$ with remaining water molecules $\mathcal{R}$ in kcal/mol			
**Water**	$E_{int}(\mathcal{W}n,\mathcal{R})$	**mol-FAMSEC**	$V_{XC}(\mathcal{W}n,\mathcal{R})$	**% of E_{int}**
aad $\mathcal{W}1$	-99.41	-58.11	-68.66	69.07
add $\mathcal{W}2$	-93.67	-53.84	-67.12	71.65
aad $\mathcal{W}3$	-65.96	-38.81	-48.00	72.78
add $\mathcal{W}4$	-72.50	-41.79	-53.10	73.24
aad $\mathcal{W}5$	-72.60	-43.79	-51.07	70.35
add $\mathcal{W}6$	-69.79	-41.82	-52.62	75.39
Average:	-79.0	-46.4	-56.8	72.1
St. Dev.:	13.9	7.7	8.8	2.2

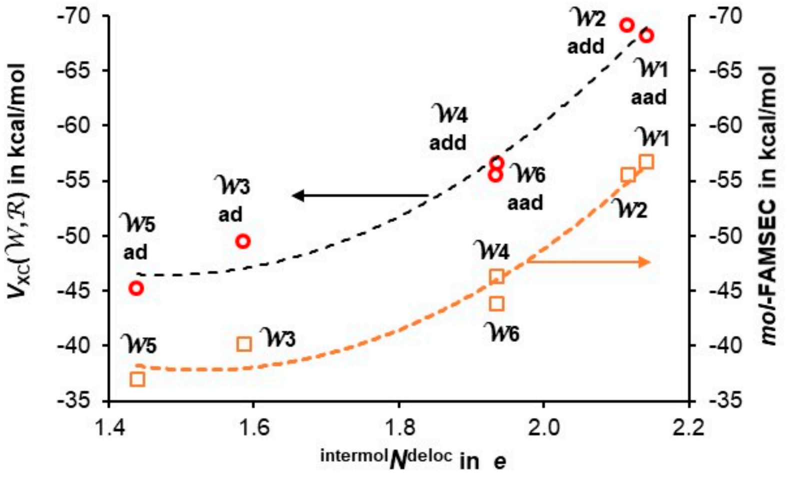

Figure S1. Trends between intermolecularly delocalized electrons by indicated water molecules in the cage water cluster, the $^{intermol}N^{deloc}$ term, and (i) circles - the quantum (exchange-correlation) term of the interaction energy between a water molecule and remaining waters in cage, the $V_{XC}(\mathcal{W},\mathcal{R})$ term, and (ii) squares – the *mol*-FAMSEC energy term that quantifies water's contribution to stability of a cluster.

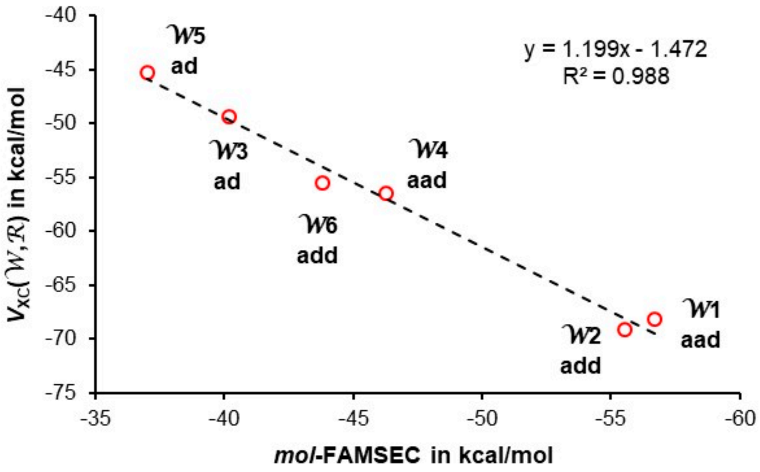

Figure S2. A trend between the *mol*-FAMSEC energy term and the quantum (exchange-correlation) term of the interaction energy between a water molecule and remaining waters in cage cluster, the $V_{XC}(\mathcal{W},\mathcal{R})$ term.

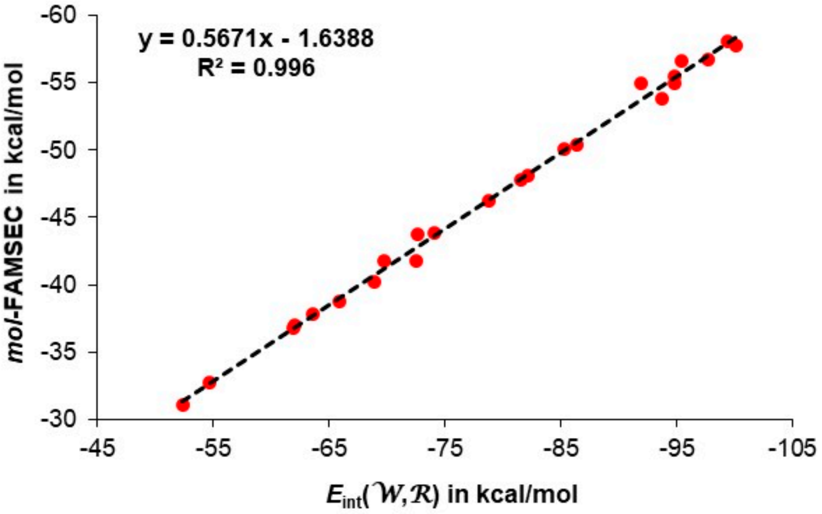

Figure S3. Correlation between interaction energy a water molecule is involved in with remaining waters in a cluster, the $E_{\mathrm{int}}(\mathcal{W},\mathcal{R})$ term, and *mol*-FAMSEC quantifying contribution made by each molecule to stability of a cluster. Full circles represent data obtained for water molecules in all 3D hexamers examined in this work.

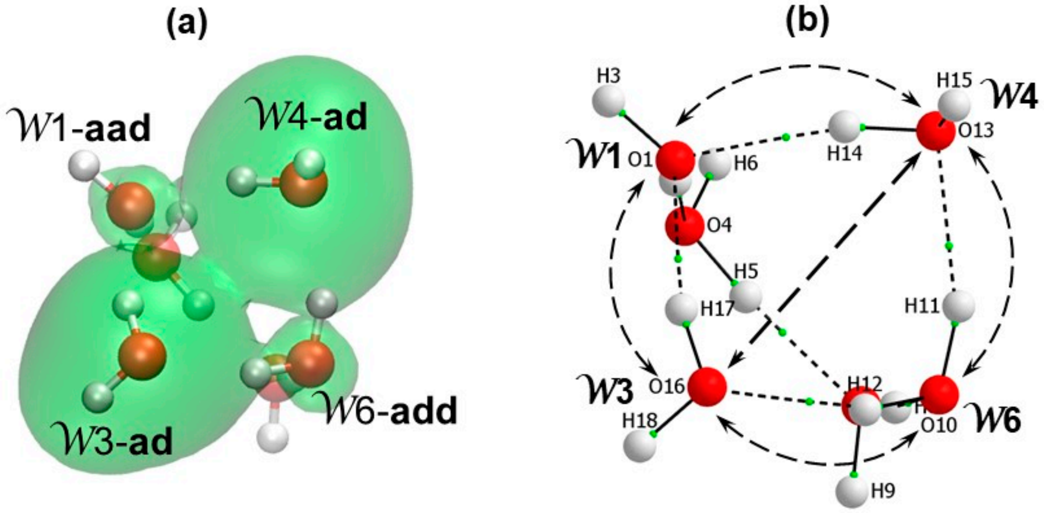

Figure S4. (a) Visual presentation of FALDI 3D density distribution between waters $\mathcal{W}$3-**ad** and $\mathcal{W}$4-**ad** of the bag hexamer. It shows how classical intermolecular H-bonding between $\mathcal{W}$4···$\mathcal{W}$1···$\mathcal{W}$3 and $\mathcal{W}$4···$\mathcal{W}$6···$\mathcal{W}$3 serves as *a unique, 'privileged' and the most effective mode of transport via density bridges (classical bond paths) for electrons delocalized predominantly by O-atoms of waters throughout a molecular system.* In addition, this picture shows that O-atoms can also share density directly (i.e. through-space) without a classical bond path with associated bond critical point. The 3D density distribution is shown using isosurfaces of 0.000025 a.u. (b) Molecular graphs showing schematic presentation of *e*-delocalization between $\mathcal{W}$3 and $\mathcal{W}$4
